# Taxifolin Reduces Blood Pressure via Improvement of Vascular Function and Mitigating the Vascular Inflammatory Response in Spontaneously Hypertensive Rats

**DOI:** 10.3390/ijms241612616

**Published:** 2023-08-09

**Authors:** Silvia Liskova, Sona Cacanyiova, Martina Cebova, Andrea Berenyiova, Michal Kluknavsky, Andrea Micurova, Katarina Valachova, Ladislav Soltes, Iveta Bernatova

**Affiliations:** 1 Centre of Experimental Medicine, Slovak Academy of Sciences, Institute of Normal and Pathological Physiology, Sienkiewiczova 1, 813 71 Bratislava, Slovakia; silvia.liskova@savba.sk (S.L.); sona.cacanyiova@savba.sk (S.C.); martina.cebova@savba.sk (M.C.); andrea.berenyiova@savba.sk (A.B.); michal.kluknavsky@savba.sk (M.K.); andrea.micurova@savba.sk (A.M.); 2 Faculty of Medicine, Institute of Pharmacology and Clinical Pharmacology, Comenius University, Sasinkova 4, 811 08 Bratislava, Slovakia; 3 Centre of Experimental Medicine, Slovak Academy of Sciences, Institute of Experimental Pharmacology and Toxicology, Dubravska cesta 9, 841 04 Bratislava, Slovakia; katarina.valachova@savba.sk (K.V.); ladislav.soltes@savba.sk (L.S.)

**Keywords:** dihydroquercetin, isolated femoral artery, isolated thoracic aorta, spontaneously hypertensive rats

## Abstract

The effect of a 10-day-long treatment with taxifolin (TAX, 20 mg/kg/day p.o.) was investigated on spontaneously hypertensive rats (SHRs) with a focus on the vascular functions of isolated femoral arteries and thoracic aortas. TAX reduced blood pressure in SHRs. In femoral arteries, TAX increased acetylcholine-induced relaxation, reduced the maximal NA-induced contraction, and reduced acetylcholine-induced endothelium-dependent contraction (EDC); however, TAX had no effect on the vascular reactivity of isolated thoracic aortas. In addition, TAX elevated the total nitric oxide synthase (NOS) activity and iNOS protein expression but reduced cyclooxygenase-2 (COX2) protein expression in the tissue of the abdominal aorta without changes in *Nos2* and *Ptgs2* gene expressions. TAX also increased the gene expression of the anti-inflammatory interleukin-10 (*Il10*). In addition, in vitro studies showed that TAX has both electron donor and H atom donor properties. However, TAX failed to reduce superoxide production in the tissue of the abdominal aorta after oral administration. In conclusion, our results show that a decrease in the blood pressure in TAX-treated SHRs might be attributed to improved endothelium-dependent relaxation and reduced endothelium-dependent contraction. In addition, the results suggest that the effect of TAX on blood pressure regulation also involves the attenuation of COX2-mediated pro-inflammation and elevation of anti-inflammatory pathways.

## 1. Introduction

Hypertension is recognized as one of the most important risk factors for cardiovascular disease, which contributes to lowering the quality of life, and almost half of hypertensive patients and approximately one-third of patients with comorbidities are nonadherent to medication [1]. Thus, the consumption of food with beneficial properties has the potential to alleviate the impact of hypertension and cardiovascular complications on the health status of patients thereby presenting a viable means to improve their quality of life. Flavonoids are natural compounds that have antioxidant and anti-inflammatory properties. Natural flavanonol taxifolin (TAX), also known as dihydroquercetin, has been shown to scavenge reactive oxygen species and chelates transition metal ions, such as iron and copper ions [2]. Furthermore, the beneficial effects of TAX involve the suppression of inflammation and increases in nitric oxide (NO) bioavailability in various experimental models [3]. It was shown that in activated macrophages, TAX inhibits lipopolysaccharide-induced prostaglandin E2 (PGE_2_) production [4]. After the start of the COVID-19 pandemic, TAX gained attention when it was recognized as a putative inhibitor of SARS-CoV-2 main protease M^pro^ [5].

To investigate more detailed mechanisms of TAX on the vascular function in hypertension, we used isolated aortas and femoral arteries of spontaneously hypertensive rats (SHRs) in which endothelial dysfunction is well documented [6]. The aorta, as an elastic artery, plays a significant role in maintaining blood pressure and heart function [7]. Previously, we confirmed that thoracic aortas participate in blood pressure (BP) control via increased NO-dependent vasorelaxation, as well as increased release of cyclooxygenase (COX)-produced vasoconstrictors in SHRs [8]. Femoral arteries are important for maintaining proper circulation into the lower limbs. The regulation of the vascular tone of arteries involves the release of NO during endothelium-dependent acetylcholine (ACh)-induced relaxation [8,9], as well as endothelium-dependent contraction (EDC) [10]. Moreover, diverse endothelium-derived factors [11] contribute to the regulation of vascular tone. It was shown that ACh, in addition to relaxation, also induces the release of endothelium-derived contracting factors, mainly in SHRs [10] but also in elderly normotensive rats [12]. In addition, SHRs have been shown to exhibit increased levels of pro-inflammatory cytokines, which suggests that they may be a useful model for studying the relationship between inflammation and hypertension [13,14]. Despite continuous disputes, studies support the hypothesis that inflammation may be causally related to the development of elevated BP in humans [15]. Targeting suitable cross-sections in the inflammation–hypertension relationship to maximize the benefits without adverse effects remains elusive at the population level, since the blood-pressure-lowering effects of anti-inflammatory agents appear to be limited to patients with active pro-hypertensive inflammatory mechanisms, such as altered endothelial function, vascular remodeling, or endocrine regulation [16].

In this study, we evaluated the effect of 10-day-long TAX treatment in SHRs. We hypothesized that the contractile function of these arteries of SHRs might be mitigated by the use of TAX, while ACh-induced endothelium-dependent relaxations might be improved via the modulation of nitric oxide production, oxidative state, and/or inflammation. In addition, we investigated the mechanism of the antioxidant effects of TAX using in vitro methods and after oral treatment in rats.

## 2. Results

### 2.1. Basic Characteristics of Experimental Animals

The body weights, systolic BP, and heart rate of the SHRs involved in the experiment were 302 ± 3 g, 173 ± 3 mmHg, 500 ± 11 bpm (*n* = 16), and there were no significant differences in the individual parameters among the groups at the beginning of the experiment. The changes in the body weight, BP, and heart rate at the end of the experiment are shown in Table 1.

### 2.2. Vascular Responses of Isolated Femoral Arteries

#### 2.2.1. NA-Induced Contraction of Isolated Femoral Arteries

**Figure 1:**
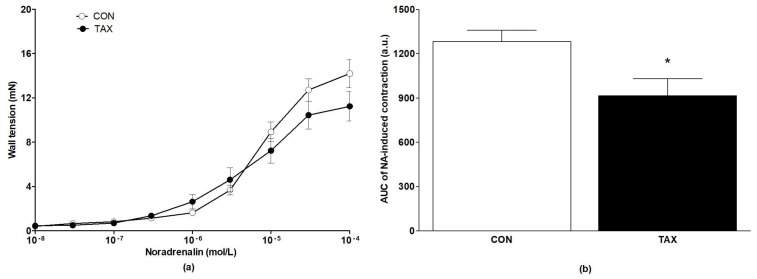
(**a**) NA-induced concentration–response curves of femoral arteries; (**b**) AUC of the NA-induced contractions of the femoral arteries of the CON and TAX groups. The results are the mean ± SEM. * *p* < 0.05 vs. CON. AUC, area under the curve; a.u., arbitrary units; NA, noradrenaline; CON, control group; TAX, taxifolin-treated group.

The sensitivity (expressed as the EC_50_ value) to NA was not affected by TAX treatment (Table 2), but the maximal contraction induced by NA tended to be reduced (Table 2, Figure 1a). This reduction was significant when calculated as the AUC of the NA-induced contraction of femoral arteries (Figure 1b).

#### 2.2.2. ACh-Induced Endothelium-Dependent Relaxation and Endothelium-Dependent Contraction of Isolated Femoral Arteries

**Figure 2:**
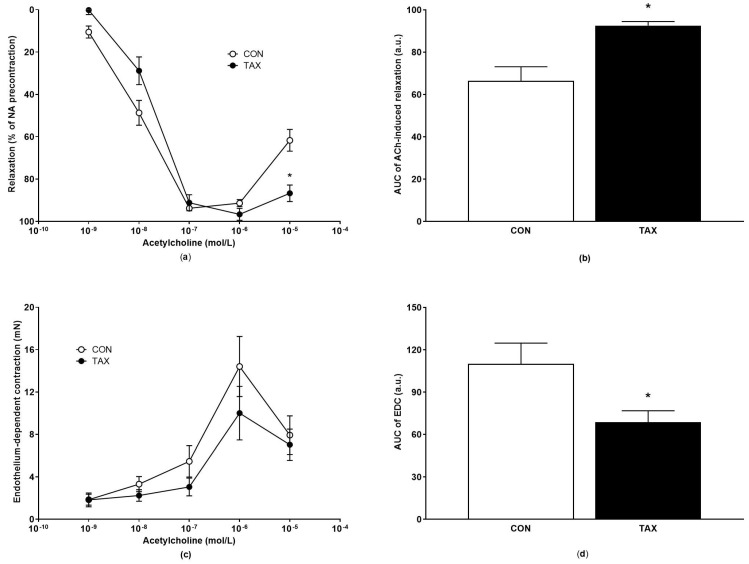
(**a**) ACh-induced concentration-response relaxation curves of femoral arteries; (**b**) AUC of ACh-induced relaxation of femoral arteries of the CON and TAX groups; (**c**) ACh-induced endothelium-dependent concentration-response contraction curves of femoral arteries; (**d**) AUC of ACh-induced endothelium-dependent contraction of femoral arteries of the CON and TAX groups. The results are the mean ± SEM. * *p* < 0.05 vs. CON. ACh, acetylcholine; EDC, endothelium-dependent contraction; AUC, area under the curve; a.u., arbitrary units; CON, control group; TAX, taxifolin-treated group.

The maximal endothelium-dependent relaxation induced by cumulative concentrations of ACh did not significantly change between groups when calculated using Hill’s equation (Table 2). At a concentration of 10^−5^ mol/L ACh, the relaxation significantly increased with TAX treatment compared to the CON group (61.7 ± 5.1% CON group vs. 86.7 ± 3.9%, *p* = 0.033, Figure 2a), suggesting the reduced release of endothelium-derived contracting factors in the TAX-treated rats. The AUC of ACh-induced relaxation was elevated in the TAX group, showing improved ACh-induced relaxations in this group (Figure 2b). Reduced ACh-induced contractions were confirmed using the calculation of the AUC of the individual concentration–response curves (Figure 2c,d). The EDC significantly decreased in the TAX group (Figure 2c,d).

### 2.3. Vascular Responses of Isolated Thoracic Aortas

The E_max_ and EC_50_ values of NA-induced contractions, as well as ACh-induced relaxations of thoracic aorta, are shown in Table 3.

**Figure 3:**
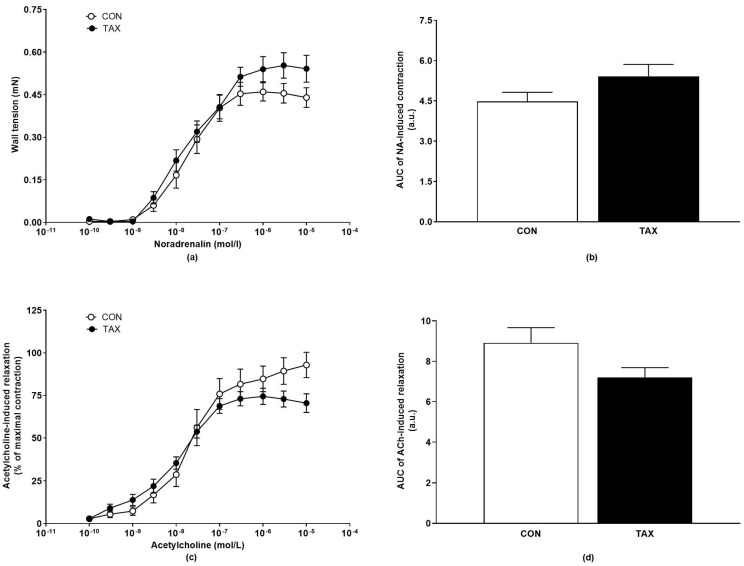
(**a**) NA-induced concentration-response curves of thoracic aortas; (**b**) AUC of NA-induced concentration-response curves of thoracic aortas; (**c**) endothelium-dependent relaxation of acetylcholine-induced concentration–response curves and (**d**) AUC of acetylcholine-induced relaxation of thoracic aortas of the CON and TAX groups. The results are the mean ± SEM. AUC, area under the curve; a.u., arbitrary units; NA, noradrenaline; CON, control group; TAX, taxifolin-treated group.

There were no significant differences in the NA-induced contractions of the isolated thoracic aortas, as well as in the AUCs of the NA-induced concentration-response curves among the groups (Figure 3a,b). The ACh-induced relaxation of the thoracic aorta was not altered significantly (Figure 3c,d).

### 2.4. Total NOS Activity and Superoxide Production in Tissue of Abdominal Aorta

**Figure 4:**
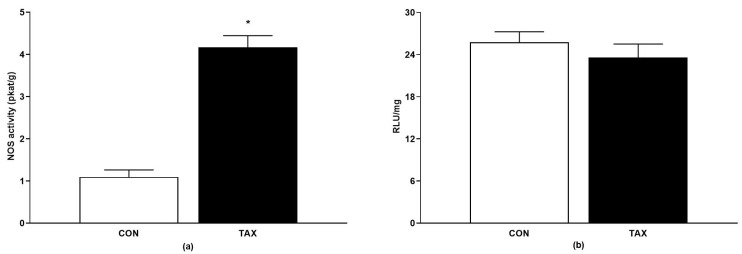
(**a**) Total NOS activity; (**b**) production of superoxide in the tissue of abdominal aorta of the CON and TAX groups. The results are the mean ± SEM. * *p* < 0.05 vs. CON. NOS, nitric oxide synthase; RLU, relative luminescence units; CON, control group; TAX, taxifolin-treated group.

The TAX-treatment enhanced the total NOS activity by approximately 300% vs. the control group (CON) (Figure 4a). TAX had no effect on superoxide production in the aorta after oral treatment (Figure 4b).

### 2.5. Protein Expressions of eNOS, iNOS, and COX2 in Tissue and Gene Expression of Corresponding Genes in Abdominal Aorta

**Figure 5:**
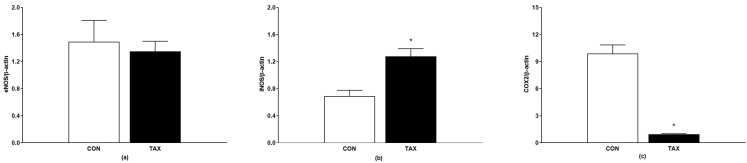
(**a**) Protein expression of eNOS; (**b**) protein expression of iNOS; (**c**) protein expression of COX2 in tissue of abdominal aorta of the CON and TAX groups. The results are the mean ± SEM. * *p* < 0.05 vs. CON. eNOS, endothelial nitric oxide synthase; iNOS, inducible nitric oxide synthase; COX2, cyclooxygenase-2; CON, control group; TAX, taxifolin-treated group.

We did not find significant differences in the protein expression of eNOS between the groups (Figure 5a). The protein expression of iNOS in the abdominal aortic tissue increased in the TAX-treated group (Figure 5b), whereas the protein expression of COX2 decreased in the tissue of the abdominal aorta of the TAX-treated rats (Figure 5c).

**Figure 6:**
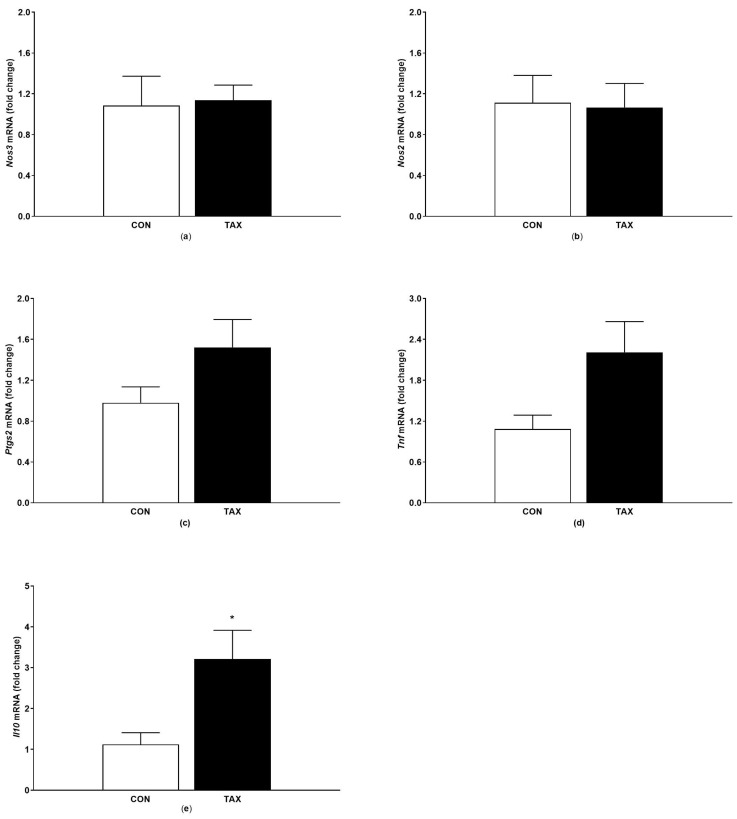
(**a**) Gene expression of *Nos3* in tissue of abdominal aorta; (**b**) gene expression of *Nos2* in tissue of abdominal aorta; (**c**) gene expression of *Ptgs2* in tissue of abdominal aorta; (**d**) gene expression of *Tnf* in tissue of abdominal aorta; (**e**) gene expression of *Il10* in tissue of abdominal aorta of the CON and TAX groups. The results are the mean ± SEM. * *p* < 0.05 vs. CON. *Nos3*, endothelial nitric oxide synthase; *Nos2,* inducible nitric oxide synthase; *Ptgs2*, cyclooxygenase-2; *Il10*, interleukin 10; *Tnf*, tumor necrosis factor alpha; CON, control group; TAX, taxifolin-treated group.

No significant differences were observed in the gene expression of *Nos3*, *Nos2* (Figure 6a,b), *Ptgs2* (Figure 6c, *p* = 0.11 vs. control), and *Tnf* (Figure 6d, *p* = 0.08 vs. control). The administration of TAX significantly increased the gene expression of *Il10* (Figure 6e).

The correlations of the gene expressions of *Nos2* and *Il10,* respectively, with *Tnf* indicate an involvement of the activation of gene expression of *Nos2* (CON: r = 0.6633, *p* = 0.0257; TAX: r = 0.9475, *p* < 0.001) and of *Il10* (CON: r = 0.9558, *p* = 0.0007; TAX: r = 0.9067, *p* = 0.0003) in all groups during the activation of *Tnf* gene expression. The correlations between the gene expressions of *Nos2* and *Il10* indicate that the gene expression of *Nos2* was activated together with the *Il10* gene expression in both groups (CON: r = 0.8013, *p* = 0.0159; TAX: r = 0.9761, *p* < 0.001). No significant correlation was observed between the gene expressions of *Ptgs2* and *Tnf*, suggesting that neither *Ptgs 2* was activated during the *Tnf* gene activation nor *Il10* gene.

### 2.6. Evaluation of Antioxidant Effects of TAX In Vitro

**Figure 7:**
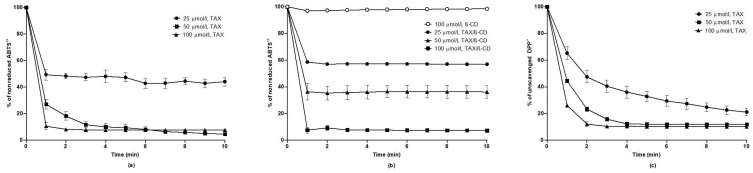
(**a**) Taxifolin reduction of ABTS•+ reagent when taxifolin was dissolved in aqueous ethanol at final drug concentrations of 100 µmol/L (triangle), 50 µmol/L (square), and 25 µmol/L (circle); (**b**) reduction of ABTS^•+^ using HP-β-CD solution at a concentration of 100 µmol/L (open circle) and taxifolin dissolved in deionized water with the complexation agent HP-β-CD at final drug concentrations of 100 µmol/L (square), 50 µmol/L (triangle), and 25 µmol/L (circle); (**c**) time-dependent changes in the percentage of unscavenged DPP^•^ reagent using taxifolin dissolved in ethanol at final drug concentrations of 100 µmol/L (triangle), 50 µmol/L (square), and 25 µmol/L (circle). The results are the mean ± SEM. ABTS^•+^, 2,2-azinobis-(3-ethylbenzothiazoline-6-sulfonic acid) diammonium salt; DPP^•^, 2,2-diphenyl-1-picrylhydrazyl radical; β-CD, 2-hydroxypropyl-β-cyclodextrin.

As shown in Figure 7a, TAX (dissolved in 96% ethanol) at concentrations 50 and 100 µmol/L was very potent at reducing ABTS^•+^ reagent. The percentage of the nonreduced ABTS^•+^ was about 4% after a 10 min measurement. After the addition of TAX at its lowest concentration 25 µmol/L, ~44% of the ABTS^•+^ remained nonreduced.

The results in Figure 7b show that HP-β-CD itself does not have electron donor properties. In contrast, within 10 min the TAX/HP-β-CD guest–host complex at concentrations of 25 µmol/L, 50 µmol/L, and 100 µmol/L, the fractions of 57%, 36%, and 7% of ABTS^•+^ remained nonreduced, respectively. We suppose that e^−^_aq._ (water-solvated electron) from TAX freely diffuses in the aqueous milieu and interacts with the ABTS^•+^ reagent, which is out of the HP-β-CD cavity.

Furthermore, TAX dose-dependently scavenged the DPP^•^ reagent. The percentages of unscavenged DPP^•^ after the 10 min measurement were approximately 21%, 12%, and 10% when examined at TAX concentrations of 25 µmol/L, 50 µmol/L, and 100 µmol/L, respectively (Figure 7c). These results show that TAX has both electron donor and H atom donor properties.

## 3. Discussion

The main findings of this study are that (1) TAX reduced BP in SHRs; (2) TAX reduced the NA-induced contraction of femoral arteries; (3) TAX improved the endothelium-dependent ACh-induced relaxation; and (4) it reduced the ACh-induced EDC. These beneficial effects of TAX were rather mediated by augmented NOS activity and reduced COX2 protein expression than by direct antioxidant effects of TAX, since long-term oral TAX-treatment did not change the superoxide production in contrast to its in vitro antioxidant effects.

In previous studies, TAX treatment attenuated markers of oxidative stress, inflammation, and apoptosis in the liver of cyclophosphamide-injected mice [17]. The antioxidant effects of TAX were found previously in N^ω^-nitro-L-arginine methyl ester (L-NAME)-induced hypertension [18]. In that study, TAX (100 µg/kg/day) reduced the amount of reactive oxygen and nitrogen species in the rat aorta that increased as a result of L-NAME intake [18]. It is of interest, that we did not find decreased superoxide production in the aorta after 10 days of TAX treatment, which in contrast to our in vitro findings, which showed that TAX has both electron donor and H atom donor properties. This suggests that metabolization of TAX after oral treatment may considerably reduce its antioxidant properties in vivo. In addition, these findings suggest that antioxidant properties of TAX are not a main mechanism in the decrease in BP in TAX-treated SHRs.

Regarding blood pressure, our findings of reduced BP in SHRs are consistent with previous research showing that TAX has antihypertensive effects in this rat strain [19]. However, it should be noted that TAX was found to be ineffective in early onset hypertension, as demonstrated in a study by Slashcheva et al. [20]. In order to disclose TAX-mediated mechanisms involved in the BP reduction, we focused on vascular mechanisms in two different arteries: thoracic aorta and femoral artery. We found that TAX reduced the NA-induced contraction of the femoral arteries without changes in their sensitivity to NA, which was associated with a reduction in ACh-induced EDC and improved ACh-induced endothelium-dependent relaxation. The reduction in EDC by TAX treatment could be mediated via the inhibition of angiotensin-converting enzyme (ACE), since it was shown that TAX reduced the activity of ACE [18]. In contrast, TAX did not alter aortic function, i.e., TAX did not influence the overall relaxation, contraction, or the sensitivity and maximal NA-induced contraction in the aorta. This points to differences in the mechanisms involved in the vascular regulation of the aorta and femoral artery, supposedly due to the differences in their structure, the role of NO in their functions [21,22], or the diverse ion channel involvement [23]. A reduction of NA-induced contraction after TAX administration was also observed in the rat portal vein, where TAX did not modify the basal venous tone but reduced the amplitude of contractions induced by KCl and NA [24]. In another study using aorta, TAX provided antispasmodic and spasmolytic action towards the NA-induced contraction of aortic rings deprived of perivascular adipose tissue; however, the preservation of perivascular adipose tissue mitigates both actions [25], which is in agreement with our observations.

Another mechanism involved in the regulation of the vascular tone involves endothelium-dependent NO-mediated relaxation, which is induced by ACh. In our study, we found that NOS activity increased four-fold after TAX administration, which indicated elevated NO bioavailability followed by increased endothelium-dependent relaxation. A study in which TAX was included in a red rice extract showed that TAX was the major active substance that exhibited strong endothelium-dependent vasorelaxant properties that were, at least, partially mediated via NO-dependent signaling through the phosphoinositide 3-kinase/eNOS pathway [26]. However, in our study, the elevation of NOS activity was not associated with elevated eNOS protein expression but with elevated iNOS protein expression without any change in the corresponding *Nos* genes, suggesting a TAX effect upon translation and/or activation of individual NOS isoenzymes. Furthermore, it is worthy to note that the depressor effects of TAX may result from a reduction in shear stress-induced phospholipase A2/COX2-mediated signaling [11]. Indeed, in the TAX-treated rats, the protein expression of COX2 significantly decreased.

Regarding COX2, its protein expression decreased in the aorta of the TAX-treated group compared to the control group, suggesting a reduction in the prostaglandin-dependent inflammatory response after TAX treatment in this study. As mentioned before, iNOS protein expression was elevated in the TAX-treated rats suggesting that COX2 and iNOS might not be activated by the same TAX-mediated triggers or might not be activated at the same time. Contrary to our observations, an in vitro study that used lipopolysaccharide-stimulated HaCaT cells, TAX was shown to inhibit iNOS and COX2 protein expression and decreased NO levels and PGE_2_ levels in these cells [27]. Another in vitro study showed that mRNA, as well as the protein levels of iNOS and COX2, were reduced together with the mRNA and protein levels of vascular endothelial growth factor and tumor necrosis factor alpha (TNF-α) in RAW264.7 cells stimulated by lipopolysaccharide [28]. These data suggest that while in in vitro conditions, TAX exerted anti-inflammatory properties, and such an effect on iNOS was not present during the 10-day treatment in vivo.

On the other hand, the expression of the anti-inflammatory *Il10* gene was found to be upregulated in the TAX-treated group in our study, while no changes in the expression of pro-inflammatory genes (*Nos2*, *Ptgs2*, and *Tnf*) were found. This suggests the possible involvement of TAX in anti-inflammatory mechanisms that may also considerably alter vascular function. Similar to our findings, the serum level of IL-10 increased, and the plasma level of TNF-α was reduced after 14 days of TAX-treatment in a model of dextran sulfate sodium-induced colitis in mice (when compared to mice with colitis induced by dextran sulfate sodium) [29]. In addition, the protein expression of TNF-α was downregulated, while IL-10 expression was upregulated with 14 days of TAX treatment in thioacetamide-induced hepatic encephalopathy [30]. Based on the abovementioned results, it is plausible to propose that treatment with TAX for a duration exceeding 10 days may result in a more pronounced inhibition of inflammatory pathways at the protein level than at the corresponding mRNA level. Moreover, Tinsley et al. [31] demonstrated that intraperitoneal administration of IL-10 normalized blood pressure and improved endothelial dysfunction in pregnant deoxycorticosterone acetate/saline-treated rats. This suggests that IL-10 may alter blood pressure regulatory mechanisms at the level of vascular function.

Taken together, we found that TAX reduced NA-induced contraction and improved ACh-induced relaxation of femoral arteries. TAX also increased NOS activity and *Il10* mRNA expressions and reduced COX2 protein expression in abdominal aorta, which was associated with reduction of blood pressure in SHRs. Altogether, our results confirm the depressor effect of TAX associated with improved NO bioavailability and anti-inflammatory effects despite the lack of a TAX effect on superoxide production.

## 4. Materials and Methods

The SHRs were obtained from the accredited breeding facility of the Department of Toxicology and Laboratory Animal Breeding, Centre of Experimental Medicine, Slovak Academy of Sciences, Dobra Voda, Slovak Republic, at the age of 16 weeks. The rats were housed under a 12 h light/12 h dark cycle at a constant humidity (45–65%) and temperature (20–22 °C), with free access to standard laboratory rat chow (Altromin Spezialfutter 1324P, Lage, Germany) and drinking water or TAX solution (in TAX-treated group) ad libitum.

### 4.1. Description of Experimental Model and Blood Pressure Measurement

The SHRs were divided into control (CON; *n* = 8) and taxifolin-treated group (TAX; *n* = 8) and kept to two rats per cage. (±)-Taxifolin (Cayman Chemicals, Ann Arbor, MI, USA) was dissolved in drinking water and administered at a dose of approximately 20 mg/kg/day p.o. in drinking water for 10 days. The calculated dose of TAX was dissolved in drinking water based on the body weight of both rats in the cage and their daily drinking volume. The average concentration of TAX in the bottle was approximately 0.3 mg/mL (depending on BW and drinking volume). TAX was prepared fresh daily. In accordance with the application of the 3Rs rule (reduce, reuse, and recycle), the control rats in this study served as a part of the control group in the study by Berenyiova et al. [32], and all rats in this study (including TAX treated) had the Alzet^®^ mini-osmotic pumps, model 2002 (containing 10% dimethyl sulfoxide in isotonic saline) implanted subcutaneously on the dorsum four days before the start of oral TAX treatment [32].

Systolic BP and heart rate (HR) were measured using tail-cuff plethysmography (MRBP, IITC Life Science Inc., Los Angeles, CA, USA). The rats were trained for tail-cuff blood pressure measurements over three consecutive days before the mini-osmotic pumps’ implantation. Basal body weight, BP, and HR were measured one day before the start of TAX treatment. End values were determined one day before the rats were decapitated under brief CO_2_ anesthesia.

### 4.2. Assessment of Vascular Responses

Carefully isolated fresh segments of femoral arteries with intact endothelium were placed in a Mulvany–Halpern isometric myograph (Dual Wire Myograph system *410A*, Danish Myo Technology A/S, Aarhus, Denmark) filled with modified Krebs–Henseleit (KH) solution (in mmol/L: 119 NaCl, 4.7 KCl, 1.17 MgSO_4_·7H_2_O, 25 NaHCO_3_, 1.18 KH_2_PO_4_, 0.03 Na_2_EDTA, 2.5 CaCl_2_·2H_2_O, 5.5 glucose, 37 °C, pH 7.4) and bubbled with a mixture of 95% O_2_ and 5% CO_2_. The inner arterial diameter of the femoral arteries was set to 90% of the diameter predicted for the pressure of 100 mmHg in the wire myograph. The normalization procedure involves normalization according to the length of the segment, and it was performed according to the normalization procedure in the manual. The viability of isolated femoral arteries was tested using a depolarizing solution (125 mmol/L K^+^, NaCl was exchanged for an equimolar concentration of KCl) for 2 min. The experimental protocol was carried out after wash out as described in [33,34]. Briefly, arterial contractions were induced by cumulative concentrations of NA (10^−8^ to 10^−4^ mol/L) and then ACh-induced relaxations (10^−9^ mol/L to 10^−5^ mol/L) of NA-precontracted vessels were measured and expressed in mN. The ACh-induced relaxations were expressed as the % of maximal NA-induced contraction. The ACh-induced EDCs were calculated according to Liskova et al. [10]. This approach was developed to measure the EDC under physiological conditions, since under physiological conditions it is not possible to achieve conditions without the traces of NA.

The thoracic aortas were isolated, cleaned from the surrounded adipose and connective tissues, cut into 5 mm long rings, and vertically fixed using two stainless-steel wire triangles on one side of the triangle placed into the lumen of the thoracic aortas. While the bottom triangle was immovably fixed, the upper wire triangle was connected to isometric tension sensors (FSG-01, MDE, Budapest, Hungary), and changes in tension were registered with an NI USB-6221 AD converter (MDE, Budapest, Hungary) and S.P.E.L. Advanced Kymograph software v3.97 (MDE, Budapest, Hungary). The resting tension of 1 g was applied, and the rings were stabilized over a 45 to 60 min equilibration period to avoid nonspecific stress relaxation. The experimental protocol was described in detail previously [35]. Briefly, the organ chambers were filled with oxygenated (95% O_2_ and 5% CO_2_, 37 °C) Krebs solution containing 118 NaCl mmol/L, 5 KCl mmol/L, 25 mmol/L NaHCO_3_, 1.2 mmol/L MgSO_4_, 1.2 mmol/L KH_2_PO_4_, 2.5 mmol/L CaCl_2_, 11 mmol/L glucose, and 0.032 mmol/L Ca-Na_2_EDTA. ACh (10^−5^ mol/L) was applied on the NA (10^−6^ mol/L)-precontracted aortic rings to test the integrity of the endothelium and the contractile abilities of the smooth muscle cells. Concentration-dependent contractile responses were determined by applying cumulative concentrations of NA (10^−10^–10^−5^ mol/L). The contractile responses were expressed as the developed tension in grams and normalized according to the length (mm) of the particular ring preparation. After reaching the steady state of NA-induced contraction, cumulative concentrations of ACh were applied (10^−10^–10^−5^ mol/L). The rate of relaxation was calculated as a percentage of the maximal NA-induced contraction. After a washing out period, thoracic aortas were precontracted with NA (10^−6^ mol/L).

From individual concentration–response curves, the maximal NA-induced contraction (E_max_) and concentration producing the half-maximum of NA responses (EC_50_) were calculated and expressed as the negative logarithm of NA molar concentration according to Hill’s equation [36]. The endothelium-dependent vasorelaxation was determined on the NA-precontracted aortic rings, and E_max_ and EC_50_ were calculated according to Hill’s equation [36]. The areas under the curve (AUCs) were calculated from the concentration-dependent curves of the ACh-induced relaxation or ACh-induced contraction and NA-induced contraction.

### 4.3. Protein Expression

The protein expressions of endothelial NOS (eNOS) and inducible NOS (iNOS) isoforms and cyclooxygenase-2 COX2 were determined in the aorta with Western blot analysis, as described in detail [37,38]. Briefly, tissue samples of the aorta were homogenized in lysis buffer containing protease inhibitor cocktail (Sigma-Aldrich, Steinheim, Germany) in 0.05 mmol/L Tris, pH = 7.4. Protein concentrations were determined using the Lowry assay, and tissue homogenates with 20 µg of protein were subjected to electrophoretic separation based on their size on 12% SDS polyacrylamide gel electrophoresis and immobilized on a nitrocellulose membrane with electroblotting. The membranes were blocked with 5% nonfat milk in Tris-buffer solution (pH 7.6) containing 0.1% Tween-20 (TBS-T) for 1 h at room temperature and probed with a primary polyclonal rabbit anti-endothelial NOS and β-actin (1:1000, Abcam, Cambridge, UK); rabbit polyclonal anti-inducible NOS (1:1000, BioRad, Inc., Hercules, CA, USA); and rabbit polyclonal anti-COX2 (1:1000, Proteintech, Rosemont, IL, USA) overnight at 4 °C. Antibodies were detected using a secondary peroxidase-conjugated anti-rabbit antibody (1:5000, Abcam, Cambridge, UK) or anti-mouse antibody (1:3000, Cell Signalling, Danvers, MA, USA) at room temperature for 2 h. The signals were detected using the enhanced chemiluminescence system (Amersham, UK). The protein bands were assessed and quantified using the Chemi-DocTM Touch Imagine System (Image LabTM Touch software BioRad, Inc., Hercules, CA, USA) and normalized to β-actin bands.

### 4.4. Gene Expressions

The gene expression levels of inducible NOS (*Nos2*), endothelial NOS (*Nos3*), cyclooxygenase-2 (*Ptgs2*), interleukin 10 (*Il10*), tumor necrosis factor alpha (*Tnf*), and 60S ribosomal protein L10a (*Rpl10a*, housekeeping gene) in the aorta were determined using a two-step reverse transcription quantitative polymerase chain reaction (RT-qPCR).

The total RNA was isolated using the PureZOL™ RNA Isolation Reagent (Bio-Rad, Hercules, CA, USA), according to the manufacturer’s protocols. The amount and purity of the total isolated RNA were spectrophotometrically quantified at 260/280 nm and 260/230 nm using a NanoDrop spectrophotometer (Thermo Scientific, Waltham, MA, USA). Reverse transcription was performed using 1 µg of total RNA from each sample using an Eppendorf Mastercycler (Hamburg, Germany) and an iScript-Reverse Transcription Supermix (Bio-Rad, Hercules, CA, USA), according to the manufacturer’s protocols. Gene-specific primers were designed using the PubMed program (Primer-BLAST) and database (Gene). The DNA sequences and melting temperature of the used primers, the size of the amplicons, and the reference numbers of the templates are described in Table 4.

The PCR reactions were conducted in a final volume of 20 µL containing 2 µL of 5-fold diluted template cDNA, 10 µL SsoAdvanced mix (SsoAdvanced Universal SYBR Green Supermix, Bio-Rad, Hercules, CA, USA), 1.5 µL of both forward and reverse primers (Metabion, Germany, 4 µmol/L), and 5 µL RNAase free water (Sigma–Aldrich, Steinheim, Germany). The thermal cycling conditions were as follows: (1) 95 °C for 30 s and (2) 40 cycles consisting of (a) 95 °C for 10 sec and (b) an optimal annealing temperature (depending on the selected primer, see Table 4) for 20 s. Finally, the melt curves for the amplicon analyses were constructed at 60–95 °C, 5 s/1 °C. The PCR method was performed on a CFX96 Real-Time PCR (Bio–Rad, Hercules, CA, USA) detection system and evaluated using Bio-Rad CFX Manager software 2.0 (Bio–Rad, Hercules, CA, USA). The expression of each gene was determined in the CON group (*n* = 8) and TAX group (*n* = 8). After completion of the reaction, data analysis was performed. For each sample, the Ct values of the target genes and Ct values of the housekeeping gene *Rlp10a* were used to estimate the relative change in the specific gene expression. The relative expressions of the studied genes were calculated using the 2^−ΔΔCT^ method [39].

### 4.5. Superoxide Production Measurement

Fresh samples of the aorta (cleaned of surrounding adipose tissue) were cut into approximately 10–15 mg segments, and superoxide production was determined using lucigenin-enhanced chemiluminescence. Freshly collected tissue samples (two segments per rat) were placed in 1.5 mL Eppendorf tubes containing ice-cold modified KH solution. From a freshly prepared lucigenin (Abcam, MA, USA) stock solution (5 mmol/L, dissolved in above described ice-cold KH solution), a measuring solution was prepared with diluting lucigenin stock solution with oxygenated KH solution (bubbled with a mixture of 5% CO_2_ and 95% O_2_, pH 7.4, temperature 37 °C) to a concentration of 50 µmol/L lucigenin. Individual segments were then gradually placed in the next 1.5 mL Eppendorf test tubes containing oxygenated KH solution and incubated in the dark at 37 °C for 18 min. At the same time, 1.5 mL Eppendorf test tubes containing 1 mL of fresh lucigenin measuring solution were incubated under the same conditions in duplets. After incubation, we transferred the aortic segment into Eppendorf test tubes containing 1 mL of fresh lucigenin measurement solution and chemiluminescence was immediately measured in 10 s intervals for 3 min using a GloMax^®^ 20/20 Luminometer (Promega, Southampton, UK). Chemiluminescence was also measured for a blank sample containing lucigenin measurement solution alone. Then, the average chemiluminescence values of each aorta segment (or blank) was calculated. The average difference between the chemiluminescence of the aorta segment and the blank was used as the final value. The results were expressed in terms of relative luminescence units per mg of tissue (RLU/mg of tissue).

### 4.6. Total NOS Activity Measurement

The total NOS activity was determined in crude homogenates of the aorta by measuring the formation of [3^H^]-L-citrulline from [3^H^]-L-arginine (MP Biochemicals, California, CA, USA), as described elsewhere [40]. Briefly, 50 µL of 10% homogenates were incubated in the presence of 50 mmol/L Tris-HCl, pH 7.4, 10 µmol/L [^3^H]-L-arginine (specific activity 5 GBq/mmol), 30 nmol/L calmodulin, 1 mmol/L β-NADPH, 3 µmol/L BH_4_, and 2 mmol/L Ca^2+^ in a total volume of 100 µL. After incubation at 37 °C, the reaction was stopped by the addition of 1 mL of stop buffer containing 20 mmol/L HEPES buffer pH 5.5, 2 mmol/L EDTA, 2 mmol/L EGTA, and 1 mmol/L L-citrulline. The samples were applied to Dowex 50WX-8 columns (Na^+^ form). [^3^H]-L-citrulline was measured with the Quanta Smart TriCarb Liquid Scintillation Analyzer (TriCarb, Packard, UK). NOS activity was expressed as picokatal per gram of protein (pkat/g protein). Protein levels were determined using the Lowry method.

### 4.7. Measurements of Antioxidant and Free Radical Scavenging Properties of TAX

The free radical scavenging capacity of TAX was examined using 2,2′-azino-bis-(3-ethylbenzthiazoline-6-sulphonic acid) diammonium salt (ABTS) and 1,1-diphenyl-2-picrylhydrazyl (DPPH) assays. Both methods are commonly used for the determination of the free-radical scavenging capacity of various substances. The electron donor properties of TAX can be expressed according to Reaction (1) [41]:

ABTS^•+^ + electron donating substance → ABTS + reduced substance (a free radical) (1)

We also determined the ability of TAX as a donor of the H atom according to Reaction (2) [42]:

DPP^•^ + ^•^H donating compound → DPPH + compound (i.e., a free radical) (2)

2-Hydroxypropyl-β-cyclodextrin (HP-β-CD, Sigma-Aldrich, Steinheim, Germany) was prepared as an aqueous solution (16 mmol/L). Taxifolin (4.8 mg) was added into 1 mL of the aqueous solution of 2-hydroxypropyl)-β-cyclodextrin (HP-β-CD, 16 mmol/L). The ABTS^•+^ (Fluka, Steinheim, Germany) and DPP^•^ (Fluka, Steinheim, Germany) reagent solutions were prepared as published previously in [43]. The high-purity-grade water with conductivity ≤ 0.055 µS/cm was produced using the TKA water purification system (Water Purification Systems, Niederelbert, Germany). The stock ethanolic solution of TAX, aqueous TAX/HP-β-CD guest–host complex (4 mmol/L, 2 mmol/L, and 1 mmol/L) or the aqueous HP-β-CD solution (4 mmol/L) was added to 2 mL of the ABTS^•+^ solution. The ethanolic solution of TAX (4 mmol/L, 2 mmol/L, and 1 mmol/L) was added to 2 mL of the DPP^•^ ethanolic solution. The final concentrations of the samples in the glass cuvette were 25 µmol/L, 50 µmol/L, and 100 µmol/L. The kinetics of ABTS^•+^ and DPP^•^ reduction were monitored within 10 min in triplicate measurements at wavelengths of 730 and 517 nm, respectively. The light-absorbance measurements were carried out using a UV-Vis 1800 spectrophotometer (Shimadzu, Kyoto, Japan).

### 4.8. Chemicals

The chemicals used in this study were purchased from Merck (Darmstadt, Germany) unless stated otherwise.

### 4.9. Statistical Analysis

The normality of the data was analyzed with the Kolmogorov–Smirnov test. Two-way analysis of variance (ANOVA) for repeated measures (within–between interactions) was used to determine significant differences of the arterial responses. ANOVA analyses were followed with the Bonferroni post hoc test to determine significant differences among the groups. All other data were analyzed using the Student’s t-test. In correlation analyses, the Pearson’s correlation coefficient (r) was calculated. The differences between the means were assessed as significant at *p* < 0.05. The data were expressed as the mean ± SEM. Data were analyzed using GraphPad Prism 7.0 (GraphPadSoftware, Inc., La Jolla, CA, USA) and Statistica 13.5 (StatSoft, Hamburg, Germany). The group size was calculated using a priori analysis with G*Power software v3.1 [44] based on the expected TAX effect on vascular contractility, using the following values: effect size 0.3, α-error 0.05, power 0.95, five measurements in two groups, and correlation between repeated measures was 0.65.

## 5. Conclusions

Our study pointed to the beneficial effects of TAX as documented in femoral artery by mitigation of enhanced NA-induced contraction and reduced EDC. The beneficial influence of TAX on vascular function in conditions of hypertension was confirmed by enhanced NO production and by the inhibition of the protein expression of COX2. The gene expression of anti-inflammatory *Il10* suggest the TAX-induced activation of anti-inflammatory pathway. Our results show that reduced a COX2-mediated pro-inflammatory pathway, improved NO production, and activation of anti-inflammatory processes might be responsible for the reduced BP in TAX-treated SHRs. Thus, our findings imply that the therapeutic benefits of TAX treatment for hypertensive individuals may stem from its anti-inflammatory action in vasculature rather than from its antioxidant properties.

## Figures and Tables

**Table 1:** Changes in the basic biometric parameters of the CON and TAX groups.

	CON	TAX
	*n* = 8	*n* = 8
Δ Body weight (g)	17 ± 2	12 ± 3
Δ BP (mmHg)	20 ± 6	−10 ± 4 *
Δ Heart rate (bpm)	−7 ± 27	16 ± 21

The results are the mean ± SEM. * *p* < 0.05 vs. CON. CON, control group; TAX, taxifolin-treated group; BP, systolic blood pressure; bpm, beats per minute.

**Table 2:** EC_50_ and E_max_ values calculated using Hill’s equation for NA-induced contractions and ACh-induced relaxations of femoral arteries of the CON and TAX groups.

	CON	TAX
	*n* = 8	*n* = 8
Length of the segments (mm)	1.54 ± 0.03	1.56 ± 0.03
EC_50NA_ femoral a. (−log mol/L)	5.12 ± 0.90	5.13 ± 0.11
E_maxNA_ femoral a. (mN)	14.90 ± 1.20	12.90 ± 1.20
EC_50ACh_ femoral a. (−log mol/L)	8.22 ± 0.11	8.19 ± 0.09
E_maxACh_ femoral a. (%)	83.00 ± 2.00	85.00 ± 5.00
E_maxKCl_ femoral a. (mN)	24.00 ± 0.90	25.70 ± 1.00

The results are the mean ± SEM. NA, noradrenaline; ACh, acetylcholine; EC_50_, half-effective maximal concentration; E_max_, maximal agonist-induced contraction or relaxation; CON, control group; TAX, taxifolin-treated group.

**Table 3:** EC_50_ and E_max_ values calculated using Hill’s equation for NA-induced contractions and ACh-induced relaxations of thoracic aortas of the CON and TAX groups.

	CON	TAX
	*n* = 8	*n* = 8
EC_50NA_ thoracic aorta (−log mol/L)	7.71 ± 0.12	7.65 ± 0.15
E_maxNA_ thoracic aorta (mN)	0.46 ± 0.03	0.56 ± 0.05
EC_50ACh_ thoracic aorta (−log mol/L)	7.66 ± 0.17	8.01 ± 0.06
E_maxACh_ thoracic aorta (%)	92.00 ± 7.00	75.00 ± 5.00

The results are the mean ± SEM. NA, noradrenaline; ACh, acetylcholine; EC_50_, half-effective maximal concentration; E_max_, maximal agonist-induced contraction or relaxation; CON, control group; TAX, taxifolin-treated group.

**Table 4:** Used primers pairs in the qPCR.

Gene	Forward Primer	Reverse Primer	Tm	Amplicon Size
			(°C)	(bp)
*Nos2* (NM_012611.3)	AAA CGC TAC ACT TCC AAC GC	TGC TGA GAG CTT TGT TGA GGT C	59	91
*Nos3* (NM_021838.2)	GAT CCC CCG GAG AAT GGA GA	TCG GAT TTT GTA ACT CTT GTG CT	60	105
*Tnf* (NM_012675.3)	CGT CAG CCG ATT TGC CAT TTC	TGG GCT CAT ACC AGG GCT T	60	116
*Il10* (NM_012854.2)	GGC TCA GCA CTG CTA TGT TG	GAG CAT GTG GGT CTG GCT G	59	117
*Ptgs2* (NM_017232.3)	CTA CCA TCT GGC TTC GGG AG	TGG AAC AGT CGC TCG TCA TC	60	85
*Rpl10a* (NM_031065.1)	TCC ACC TGG CTG TCA ACT TC	GGC AGC AAC GAG GTT TAT TGG	60	134

Tm, melting temperature; bp, base pairs.

## Data Availability

The data presented in this study are contained within the article and available upon request.
